# Regulation of miRNA-29c and its downstream pathways in preneoplastic progression of triple-negative breast cancer

**DOI:** 10.18632/oncotarget.14902

**Published:** 2017-01-30

**Authors:** Anjana Bhardwaj, Harpreet Singh, Kimal Rajapakshe, Kazunoshin Tachibana, Nivetha Ganesan, Yinghong Pan, Preethi H. Gunaratne, Cristian Coarfa, Isabelle Bedrosian

**Affiliations:** ^1^ Department of Breast Surgical Oncology, The University of Texas MD Anderson Cancer Center, Houston, TX, USA; ^2^ Department of Molecular and Cellular Biology, Baylor College of Medicine, Houston, TX, USA; ^3^ Department of Biology and Biochemistry, University of Houston, Houston, TX, USA

**Keywords:** miRNA-29c, TNBC, prevention, next generation RNA sequencing, DNA methylation

## Abstract

Little is understood about the *early* molecular drivers of triple-negative breast cancer (TNBC), making the identification of women at risk and development of targeted therapy for prevention significant challenges. By sequencing a TNBC cell line-based breast cancer progression model we have found that miRNA-29c is progressively lost during TNBC tumorigenesis. In support of the tumor suppressive role of miRNA 29c, we found that low levels predict poor overall patient survival and, conversely, that ectopic expression of miRNA-29c in preneoplastic cell models inhibits growth. miRNA-29c exerts its growth inhibitory effects through direct binding and regulation of TGFB-induced factor homeobox 2 (TGIF2), CAMP-responsive element binding protein 5 (CREB5), and V-Akt murine thymoma viral oncogene homolog 3 (AKT3). miRNA-29c regulation of these gene targets seems to be functionally relevant, as TGIF2, CREB5, and AKT3 were able to rescue the inhibition of cell proliferation and colony formation caused by ectopic expression of miRNA-29c in preneoplastic cells. AKT3 is an oncogene of known relevance in breast cancer, and as a proof of principle we show that inhibition of phosphoinositide 3-kinase (PI3K) activity, a protein upstream of AKT3, suppressed proliferation in TNBC preneoplastic cells. We explored additional opportunities for prevention of TNBC by studying the regulation of miRNA-29c and identified DNA methylation to have a role in the inhibition of miRNA-29c during TNBC tumorigenesis. Consistent with these observations, we found 5 aza-cytadine to relieve the suppression of miRNA-29c. Together, these results demonstrate that miRNA-29c loss plays a key role in the early development of TNBC.

## INTRODUCTION

Breast cancer is the most common cancer in women worldwide [1]. Despite the many advances in the field of breast cancer treatment, challenges still prevail in management of breast cancer patients leading to over 40,000 breast cancer-related deaths every year in the United States alone [2]. Among the reasons for this mortality is the relative resistance of triple-negative breast cancer (TNBC) to current conventional therapies, which leads to a disproportionately higher rate of death in this subset of breast cancer patients. Therefore, in order to reduce breast cancer-related deaths, it is imperative to identify TNBC subtype-specific targets for prevention. Consequently, we have focused on detailing the molecular changes that occur in the preneoplastic stages of TNBC development in order to identify opportunities for prevention.

miRNAs are small noncoding RNAs that bind and repress the translation of its mRNA targets or cause their degradation. miRNA aberrations have been widely reported to play important roles in cancers in general through modulation of several hallmarks of cancer. In breast cancer, miRNAs have been reported to play central roles as their profiles differ between breast tumors and normal breast tissue [3]. Within breast tumors, miRNA expression patterns are associated with distinct molecular breast cancer subtypes, e.g., miRNA-18a, -135b, -93, and -155 have been identified as common TNBC-specific miRNAs in a meta-analysis of 3 independent studies [4]. To address whether miRNAs play a causal role in breast cancer development, studies such as that by Croce and colleagues [3] reported let-7d, miRNA-210, and miRNA-221 to be differentially expressed between ductal carcinoma *in situ* (DCIS) and invasive breast cancer. Recently, miRNA-140 was reported to be lost in DCIS of basal-like cancers, where its downregulation was found to promote the formation of cancer stem cells in DCIS cells *in vitro* and the formation of tumors *in vivo* [5]. While these studies suggest a role for miRNAs in the later stages of tumorigenesis, namely the transition of DCIS cells to invasive breast cancer, it is not known if miRNAs play a role in the earlier, preneoplastic steps of breast cancer development. Breast cancer is thought to develop through progressive transitions from benign hyperplasia of mammary duct epithelial cells, through to atypical ductal hyperplasia (ADH), to DCIS, invasive tumor confined within the breast, followed by lymph node involvement, and, ultimately, metastasis to distant organs. We postulated that detailing the molecular portraits of the preneoplastic stages of breast tumorigenesis would provide targets for the potential prevention of TNBC. To address this goal, we performed next-generation sequencing of an MCF10A-based TNBC progression panel and identified miRNA-29c to be lost in the preneoplastic transition to ADH, which also continued through DCIS and invasive cancer. We then demonstrated that miRNA-29c plays a tumor suppressor role in the preneoplastic phase of tumorigenesis and showed that miRNA-29c inhibition of cell proliferation and colony formation is at least in part mediated by its gene targets V-Akt murine thymoma viral oncogene homolog 3 (AKT3), TGFB-induced factor homeobox 2 (TGIF2), and CAMP-responsive element binding protein 5 (CREB5). As a proof of concept, we also show evidence that targeting one of these pathways with LY294002, a small-molecule inhibitor of PI3 kinase, inhibited cell proliferation in preneoplastic cells. Finally, we also provide evidence that targeting the repressors of miRNA-29c expression revert its expression and inhibit cell proliferation in MCF10.AT1 preneoplastic cells, thus presenting novel opportunities for the prevention of TNBC.

## RESULTS AND DISCUSSION

### Next-generation sequencing to identify molecular drivers of normal-to-preneoplastic transition in TNBC

**Figure 1:**
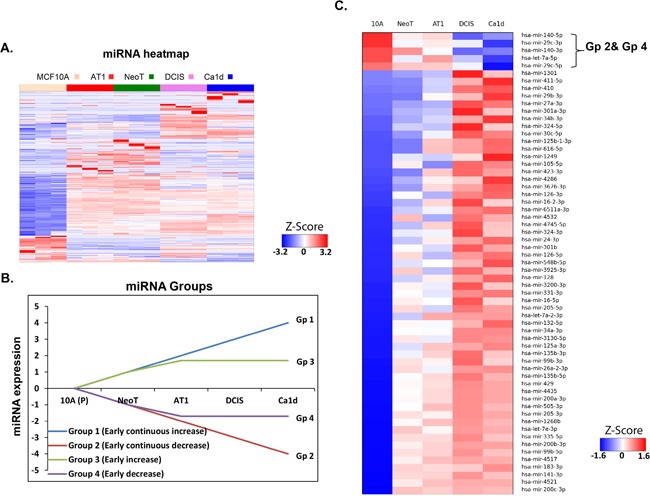
miRNA aberrations during preneoplastic transition in TNBC development **A**. Heatmap of small RNA sequencing data showing global miRNA alterations in an MCF10A TNBC progression model. MCF10A breast cancer progression model comprises of MCF10A, which represent non-cancer breast cell line; NeoT, hyperplasia; AT1, atypical hyperplasia; DCIS, ductal carcinoma *in situ*; and Ca1d, invasive carcinoma. **B**. Schematic showing 4 groups of miRNAs (focused on early changes and early-continued changes) to identify miRNAs relevant to preneoplastic transition. **C**. Heatmap of 4 groups (as described in panel B) of miRNA alterations in an MCF10A TNBC progression model derived from small RNA sequencing data.

To identify miRNA and their functional gene targets that drive the development of TNBC, we performed high-throughput miRNA sequencing of the MCF10A-based TNBC progression model. We found that more than 50% (299 of 561 miRNAs) of the miRNA alterations occurred during preneoplastic transition (normal to atypia), which indicates the enormous potential for TNBC prevention at this early stage of tumorigenesis (Figure 1A).

Because of our focus on identification of markers and targets for prevention, we were particularly interested in a group of miRNAs that change early during TNBC development. To identify such potential targets for TNBC prevention, we have focused on detailing the miRNA changes that occur in the pre neoplastic stages of development of TNBC. Therefore, we organized the miRNAs into 4 groups on the basis of how they changed across the progression continuum: early and continuous increase (Group1), early and continuous decrease (Group 2), early increase followed by plateau (Group 3), and early decrease then plateau (Group 4) (Figure 1B). Of the several hundred miRNAs that we found to be dysregulated in the progression panel, only 63 miRNA alterations mapped to any of these 4 groups (Figure 1C). We were most interested in the Group 2 and Group 4 miRNAs, as these would conversely cause the up regulation of gene and protein targets that could be more readily targeted for prevention. We identified only 5 miRNA fitting this pattern of interest: miRNA-140-5p, miR-29c-3p, miRNA-140-3p, Let-7a-5p, and miR-29c-5p. While Let -7 and miRNA-140 will be focus of our future studies, in here we selected miRNA-29c-3p (hereafter called miRNA-29c) for further study because it has been reported to be the most down-regulated miRNA in basal-like compared with luminal A [6] and other subtypes [7] of breast cancer, and it has a known role in serous ovarian carcinoma [8], a disease that is molecularly similar to TNBC [9].

### miRNA-29c is lost during TNBC progression and predicts survival in basal-like breast cancer patients in a TCGA dataset

**Figure 2:**
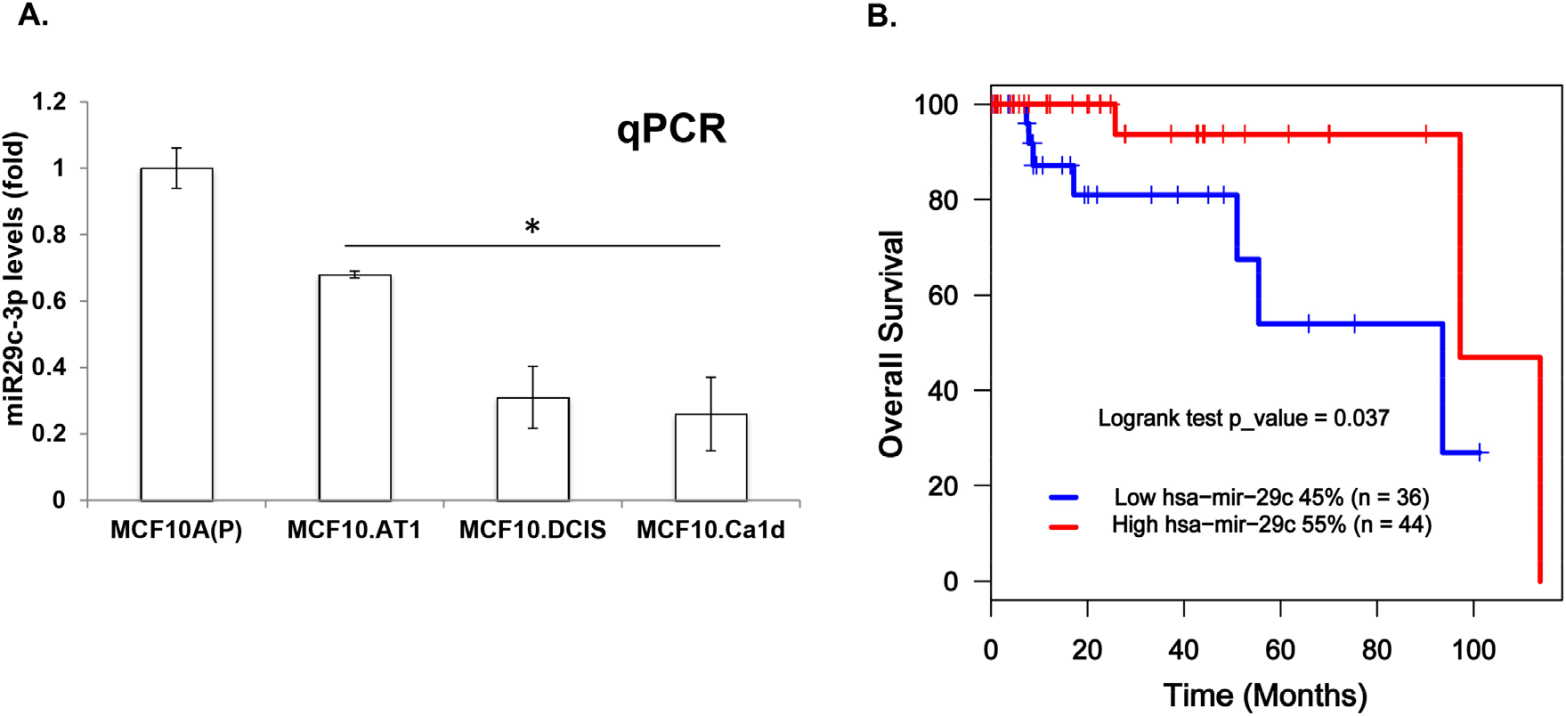
miRNA-29c is lost during TNBC progression and its low levels predict poor survival in basal-like breast cancer patients **A**. miRNA-29c-3p levels were measured by Taqman-based qPCR analysis in an MCF10A-based TNBC progression model. miRNA-29c-3p levels were normalized to RNU44 control. **B**. Kaplan-Meier curve showing overall survival (in months) in basal-like breast cancer patients expressing low vs high levels of miRNA-29c. *p<0.05.

To confirm the loss of miRNA-29c during breast cancer progression, we performed quantitative polymerase chain reaction (qPCR) and measured the levels of mature miRNA-29c in the MCF10A breast cancer progression panel. These assays confirmed a significant progressive loss of miRNA-29c (Figure 2A). Next, to evaluate the relevance of miRNA-29c to basal-like breast cancer, we mined The Cancer Genome Atlas (TCGA) basal-like breast cancer data to study its association with overall survival. These analyses showed high levels of miRNA-29c to be significantly associated with better overall survival (log rank p=0.037) in basal-like breast cancer. Specifically, median survival in patients expressing low levels of miRNA-29c was 60 months compared to 95 months in patients expressing high levels of miRNA-29c (Figure 2B). These results clearly suggest that miRNA-29c is a tumor suppressor in basal-like breast cancer and are in concordance with the findings of Nygren et al [10], who used 2 cohorts of Danish and Norwegian breast cancer patients and found high levels of miRNA-29c to be associated with longer breast cancer-related survival and with lower risk of developing distant metastasis.

### miRNA-29c inhibits proliferation and colonizing ability of preneoplastic TNBC cells

**Figure 3:**
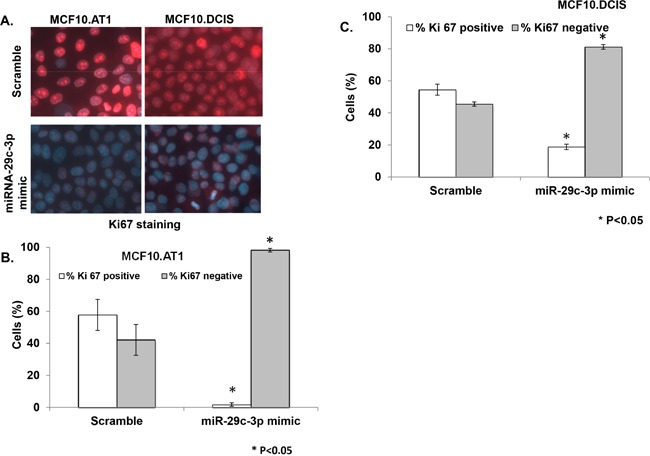
miRNA-29c-3p inhibits cell proliferation in TNBC preneoplastic and DCIS cells **A**. Ki67 immunostaining followed by immunofluorescence in MCF10.AT1 and DCIS cells that were transfected with miRNA-29c-3p mimic or scramble control mimic. Red color foci within the DAPI-stained blue nuclei indicate Ki67. **B, C**. Bar diagrams show the quantification of highly proliferative cells expressing 3 or more Ki67 foci (Ki67 positive) and minimally proliferative cells expressing 0-2 Ki67 foci (Ki67 negative) in the nuclei of MCF10.AT1 cells and MCF10.DCIS cells. * p<0.05.

While the association between miRNA-29c levels and overall survival in TCGA dataset strongly suggest the role of miRNA-29c in prognosis of basal like breast cancer; in order to understand the biologic relevance of miRNA-29c in the preneoplastic stage of tumorigenesis, we studied the effect of miRNA-29c modulation on cell proliferation in TNBC preneoplastic (AT1) and DCIS cells. Ki67 was studied as a surrogate for cell proliferation. These immunofluorescence-based assays revealed that ectopic expression of miRNA-29c in MCF10.AT1/DCIS cells significantly (P<0.05) inhibited the cell proliferation and caused a dramatic switch from highly proliferative (Ki67 positive) to minimally proliferative (Ki67 negative) cells (Figure 3). Specifically, ectopic overexpression of miRNA-29c decreased proliferation by 56% relative to the scramble control in MCF10.AT1 cells (Figure 3A & 3B). Similarly, in MCF10.DCIS cells, miRNA-29c decreased proliferation by 36% relative to the scramble control (Figure 3A & 3C). The fact that miRNA-29c caused more pronounced inhibition of cell proliferation in preneoplastic MCF10.AT1 cells relative to MCF10.DCIS cells suggests that prevention strategies based on miRNA-29c re-expression are likely to be more effective in the preneoplastic stage.

**Figure 4:**
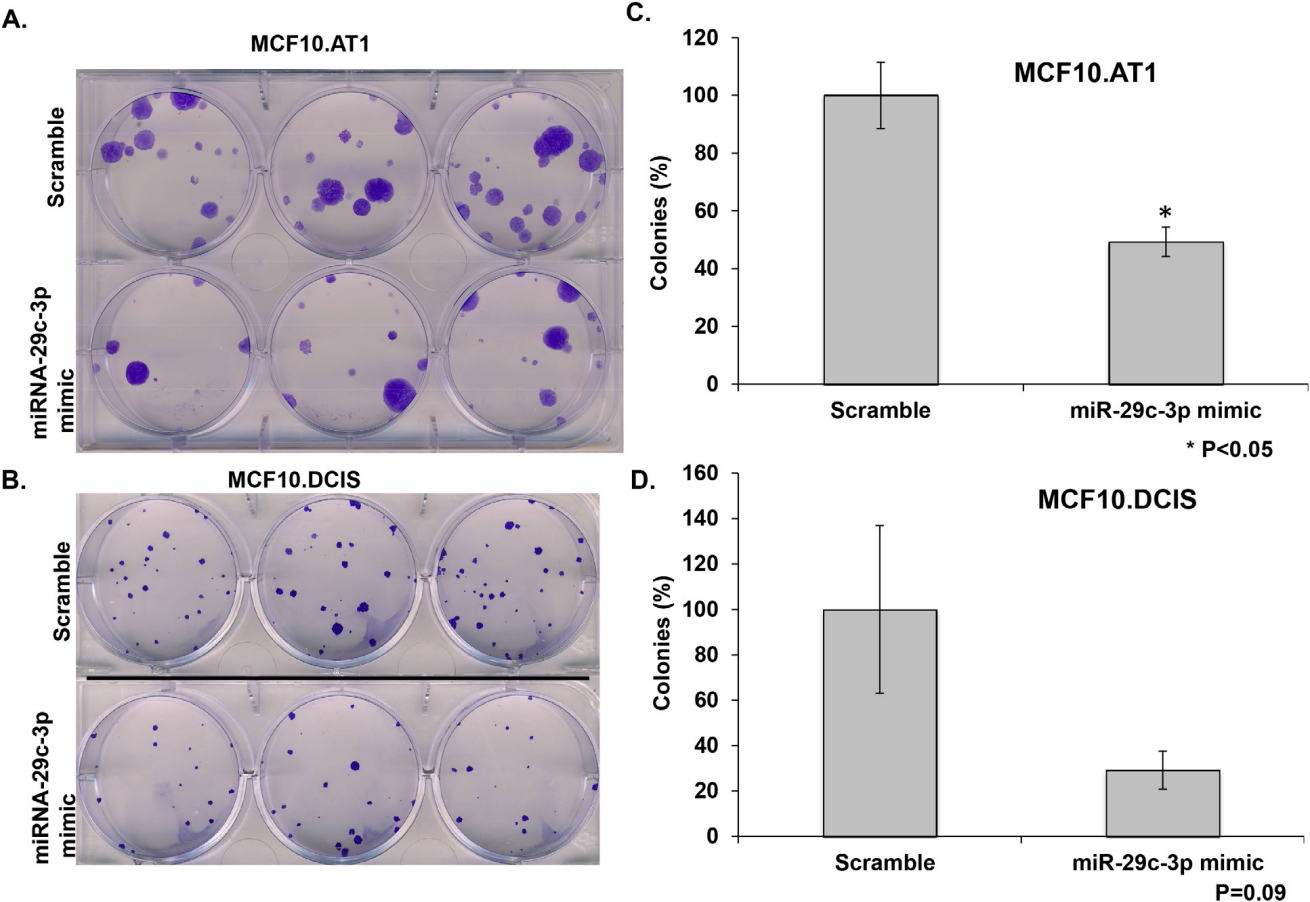
miRNA-29c-3p inhibits colony formation in TNBC preneoplastic and DCIS cells **A, B**. Methylene blue stained colonies formed by MCF10.AT1 and MCF10.DCIS cells after 12 days of transfection with miRNA-29c-3p mimic or scramble control mimic. **C, D**. Quantification of colonies formed by MCF10.AT1 cells and MCF10.DCIS cells transfected the miRNA-29c mimic or scramble mimic. Cell colonies possessing more than 50 cells were counted one colony. *p<0.05.

To test whether miRNA-29c re-expression has any impact on the ability of single cells to survive and proliferate to make a colony, we performed colony formation assays. These studies revealed that ectopic expression of miRNA-29c in MCF10.AT1 cells significantly (P<0.05) inhibited the ability of cells to colonize (Figure 4A & 4C). Specifically, ectopic expression of miRNA-29c decreased the colonizing ability of MCF10.AT1 cells by 50% relative to the scramble control. We also observed an inhibition in the colonization ability of MCF10.DCIS cells relative to the scramble control after transfection of miRNA-29c, but this reduction was not statistically significant (p=0.09, Figure 4B & 4D). These results are consistent with stronger inhibition of cell proliferation in preneoplastic cells than in DCIS cells, again suggesting that miRNA-29c has a greater biologic role in the early, preneoplastic stages of tumorigenesis.

### Integration of miRNA-29c with RNA sequencing data across the breast cancer progression continuum

**Figure 5:**
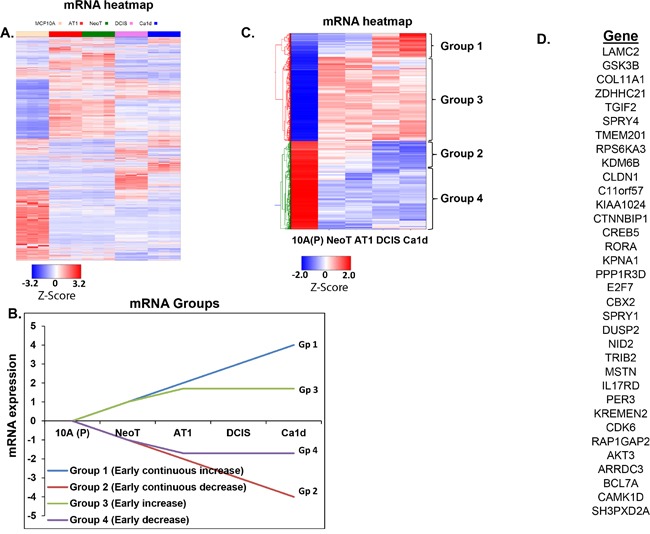
Integration of small RNA sequencing with RNA sequencing to identify gene targets of miRNA-29c-3p **A**. Heatmap generated from RNA sequencing data showing global mRNA alterations in an MCF10A TNBC progression continuum. MCF10A breast cancer progression model comprises of MCF10A, which represent non-cancer breast cell line; NeoT, hyperplasia; AT1, atypical hyperplasia; DCIS, ductal carcinoma *in situ*; and Ca1d, invasive carcinoma. **B**. Schematic showing 4 groups of mRNAs (focused on early changes and early-continuous changes) to identify genes that are altered during TNBC progression. **C**. Heatmap of mRNAs that map to the 4 groups (focused on early changes and early-continuous changes). **D**. List of 35 predicted gene targets of miRNA-29c using a set of filters, including our bioinformatics pipeline SigTerms.

To understand how miRNA-29c potentially affects the cell proliferation and colonizing ability of preneoplastic and DCIS cells, we focused on identifying its functional gene pairs and turned to our next-generation RNA sequencing data from the MCF10A breast cancer progression panel. First, we looked for any global patterns of mRNA expression during breast cancer progression. Mining of these sequencing results show that even at gene level a multitude of the gene alterations (more than 80%) occur during the normal to preneoplastic transition during breast cancer development, suggesting that molecular determination of cell fate occurs early on in the process of TNBC development (Figure 5A and 5C).

In order to identify functional gene pairs of miRNA-29c, we organized the mRNA sequencing data (Figure 5A) into the 4 progression groups defined previously (Figure 5B). Of the several thousand mRNAs that were deregulated in the progression panel (Figure 5A), several hundred-gene alterations fit the patterns of interest (Figure 5C). Interestingly, even after these groupings, about 70% of the gene alterations occurred during the normal to preneoplastic transition during TNBC development. To identify the functional gene pairs of miRNA-29c, first we obtained a list of mRNAs that significantly increased (>1.5, P<0.05) during the transition from MCF10A (P) [normal like] to MCF10.DCIS in our RNA sequencing dataset. Next, using SigTerm bioinformatics pipeline [11], we identified genes whose expression inversely correlates with miRNA-29c expression (with a false discovery threshold of <0.25) during breast cancer progression (MCF10A to DCIS transition) and that were predicted gene targets of miRNA-29c by TargetScan. This resulted in a list of 35 genes (Figure 5D).

### miRNA-29c-regulated gene targets and pathways

To validate these 35 predicted gene targets of miRNA-29c, we selected 14 genes on the basis of their predicted or demonstrated roles in cancer-relevant pathways and their drugability. The remaining 21 genes were not studied further either due to their dual roles (e.g., tumor suppressor in normal cells and oncogenic in cancer), or were not reported to be oncogenic, or were not targetable. Using qPCR-based assays, we first analyzed the baseline expression patterns of the 14 selected genes in our MCF10A-based cell line progression panel and validated more than 80% of these 14 genes (Supplementary Figure 1). As further validation, we ectopically expressed miRNA-29c in MCF10.AT1 cells by using miRNA mimics and measured the mRNA levels of these 14 genes by qPCR. These assays showed that the miRNA-29c mimic significantly repressed the expression of 9 gene targets: LAMC2, TGIF2, SPRY4, AKT3, MAP2K6, CDK6, CREB5, FOS, and E2F7 (Table 1). Next we rank ordered these genes based on their baseline expression levels during TNBC progression (Supplementary Figure 1). TGIF2 and CREB5 were top 2 genes that changed the most during MCF10A (P) to DCIS transition. AKT3 followed right after TGIF2 and CREB5, and is an oncogene of known relevance in early stages of TNBC [12]. Therefore, we further evaluated these 3 cancer-relevant gene targets (AKT3, CREB5, and TGIF2) to study their direct binding and regulation by miRNA-29c [12–14]. Since CDK6 is a known target of miRNA-29c in several cancer models [15–17], CDK6 regulation was not tested in miRNA-29c binding experiments.

**Table 1:** Quantitative PCR analysis of MCF10.AT1 cells transfected with miRNA-29c-3p mimics or scramble control for 48 h

Genes	Scramble mimic (fold)	miR-29c-3p-mimic (fold change)
LAMC2	1.00	0.46*
ZDHHC21	1.00	0.95
TGIF2	1.00	0.21*
SPRY4	1.00	0.55*
MAPK10	1.00	1.50
AKT3	1.00	0.80*
MAP2K6	1.00	0.48*
CDK6	1.00	0.20*
GSK3B	1.00	1.11
CREB5	1.00	0.50*
FOS	1.00	0.41*
RPS6KA3	1.00	0.98
E2F7	1.00	0.28*
KPNA1	1.00	1.00

Results are normalized to ribosomal protein L19 mRNA levels.

*p<0.05.

**Figure 6:**
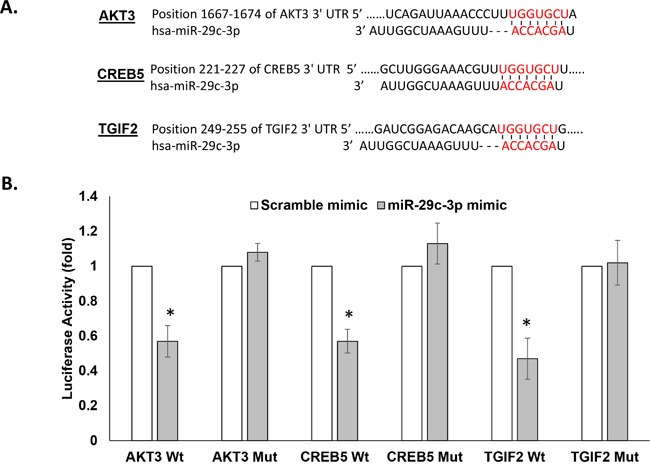
miRNA-29c-3p directly binds in the 3′UTR of AKT3, TGIF2, CREB5, and CDK6 **A**. Target Scan predicted base pairing of mature miRNA-29c-3p seed sequences in the 3′UTRs of the indicated genes. **B**. Luciferase activity in MCF10.AT1 cells transfected with the dual luciferase vector pmiRGLo containing the wild type (Wt) and mutated (Mut) miRNA-29c-3p binding sites indicated in panel A. The cells were also transfected with an miRNA-29c-3p mimic or a negative control mimic. The firefly signal reported the gene regulation by miRNA binding and the internal control renilla luciferase was used for normalizing the transfection efficiency. * p<0.05.

To test the direct binding between miRNA-29c and its gene targets, first the putative miRNA-29c binding sites from the 3′UTRs of AKT3, CREB5, and TGIF2 (Figure 6A), along with their surrounding 200-nucleotide sequences, were individually cloned into pmiRGlo, a luciferase reporter construct. Next, we co-transfected the miRNA-29c mimic with the 3′UTR luciferase reporter vector and found it to repress the luciferase activity of all 3 of these reporter vectors by ~50% (Figure 6B), indicating direct binding and regulation of these genes by miRNA-29c. Introduction of a 3-nucleotide mutation in the miRNA-29c seed sequence in either the AKT3 3′ UTR or CREB5 3′UTR or TGIF2 3′UTR abrogated this regulation (Figure 6B).

**Figure 7:**
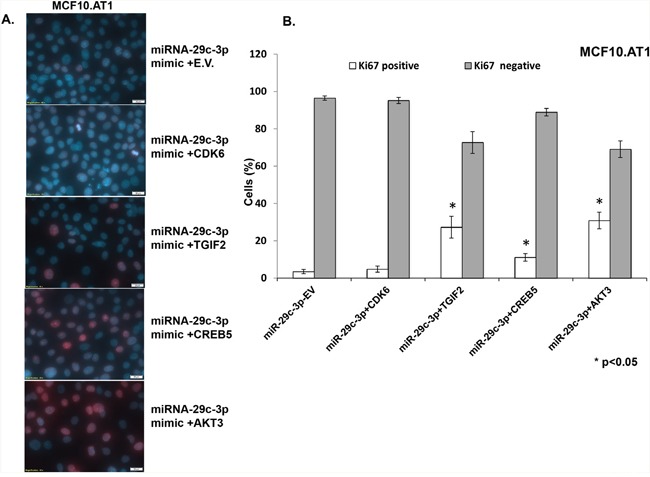
TGIF2, CREB5, and AKT3 overexpression reverts the inhibition in cell proliferation caused by forced expression of miRNA-29c-3p in TNBC preneoplastic cells **A**. Immunofluorescence images showing the cell proliferation marker Ki67 (indicated by red nuclear stain within the DAPI-stained nucleus) in MCF10.AT1 cells transfected with miRNA-29c-3p mimic in combination with TGIF2, CREB5, AKT3, CDK6, or empty expression vector (E.V.). **B**. Bar diagrams show the quantification of highly proliferative cells expressing 3 or more Ki67 foci (Ki67 positive) and minimally proliferative cells expressing 0-2 Ki67 foci (Ki67 negative) in the nuclei of MCF10.AT1 cells. * p<0.05.

**Figure 8:**
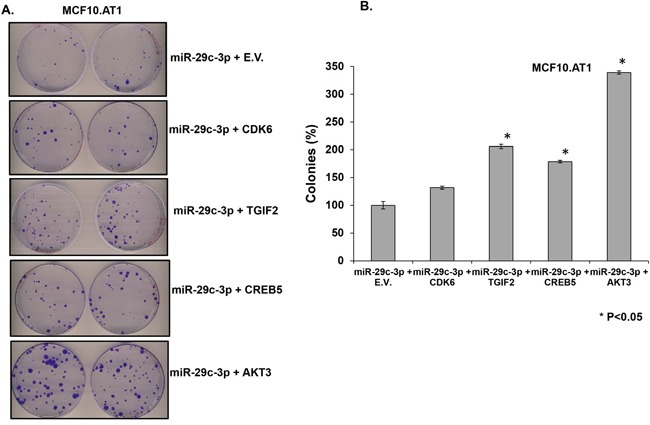
TGIF2, CREB5, and AKT3 overexpression reverts the inhibition in colony formation caused by forced expression of miRNA-29c-3p in TNBC preneoplastic cells **A**. Methylene blue-stained colonies formed by MCF10.AT1 cells after 12 days of transfection with miRNA-29c-3p mimic in combination with TGIF2, CREB5, AKT3, CDK6, or empty expression vector (E.V.). **B**. Quantification of colonies formed by MCF10.AT1 cells transfected with the miRNA-29c mimic in combination with TGIF2, CREB5, AKT3, CDK6, or empty expression plasmid. Cell colonies possessing more than 50 cells were counted as clones. * p<0.05.

**Figure 9:**
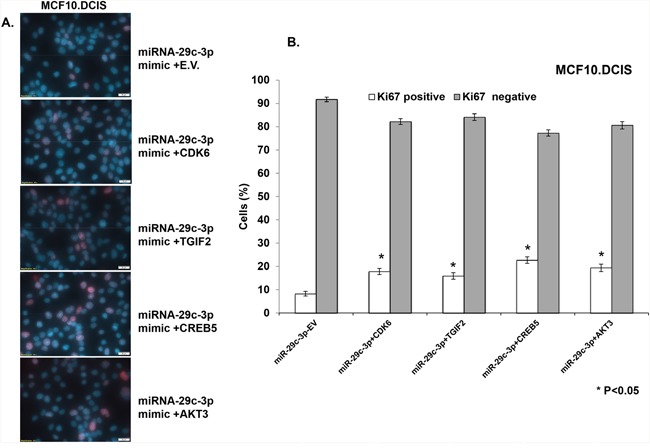
TGIF2, CREB5, and AKT3 overexpression only partially reverts the inhibition in cell proliferation caused by forced expression of miRNA-29c-3p in MCF10.DCIS cells **A**. Immuno- fluorescence images showing the cell proliferation marker Ki67 (indicated by red nuclear stain within the DAPI-stained nucleus) in MCF10.DCIS cells transfected with miRNA-29c-3p mimic in combination with TGIF2, CREB5, AKT3, CDK6, or empty expression vector (E.V.). **B**. Bar diagrams show the quantification of highly proliferative cells expressing 3 or more Ki67 foci (Ki67 positive) and minimally proliferative cells expressing 0-2 Ki67 foci (Ki67 negative) in the nuclei of MCF10.DCIS cells. * p<0.05.

Next, in order to identify effector gene targets of miRNA-29c that mediate its growth-inhibitory effects in MCF10.AT1 and MCF10.DCIS cells, we analyzed the ability of AKT3, CREB5, and TGIF2 and a known miRNA 29c gene target, CDK6, to rescue the cell-inhibitory phenotype. We co-transfected the constructs expressing AKT3, CREB5, TGIF2, and CDK6 in MCF10.AT1 and MCF10.DCIS cells that were also transfected with miRNA-29c mimics. We studied the cell proliferation ability of these cells by staining with the Ki67 antibody and the colonizing ability by performing colony formation assays. Interestingly, TGIF2, CREB5, and AKT3 overexpression resulted in a significant reversal of the inhibition in cell proliferation and colonization ability caused by the miRNA-29c mimic only (Figure 7 & 8) in preneoplastic MCF10.AT1 cells. AKT3 was most effective in rescuing both cell inhibitory phenotypes (increase in cell proliferation by 32% and colonizing ability by 3.4 fold) followed by TGIF2 (increase in cell proliferation by 24% and colonizing ability by 2.6 fold) and CREB5 (increase in cell proliferation by 8% and colonizing ability by 1.8 fold) (Figure 7 & 8). These miRNA-29c-regulated oncogenes were less effective at rescuing the inhibition of proliferation in MCF10.DCIS cells (Figure 9), highlighting again the relevance of miRNA-29c targeting primarily in preneoplastic TNBC. Interestingly, CDK6, a previously reported miRNA-29c gene target known to inhibit cell proliferation in cancer cells [17], failed to show any functional relevance in preneoplastic MCF10.AT1 cells (Figure 7 & 8). However, CDK6 marginally but significantly rescued the inhibition in cell proliferation caused by miRNA-29c in MCF10.DCIS cells (Figure 9). These findings suggest important context-dependent constraints in the miRNA regulation of target genes.

Consistent with the oncogenic roles of TGIF2 and CREB5 in rescuing the growth inhibitory phenotype of miRNA-29c (Figure 7–9), TGIF is a central mediator of β-catenin-Wnt oncogenic signaling in breast cancer [14]. Activation of β-catenin has been reported to induce TGIF expression, which in turn leads to the stabilization and accumulation of β-catenin itself and drives mammary tumorigenesis in mouse models. Higher levels of TGIF are also associated with poor prognosis in TNBC patients [14]. There is also support for β-catenin and TGF-β pathway-mediated epithelial-to-mesenchymal transition (EMT) being dependent on CREB binding protein [13], indicating possible crosstalk between TGIF2 and CREB5. AKT3 has been previously reported to be a direct target of miRNA-29c, and it inhibits cell proliferation and promotes differentiation during myogenesis [18]. Recently, AKT3 has been shown to be an oncogene of relevance in TNBC by Polyak and colleagues [12], who reported that the downregulation of AKT3 inhibits the growth of MCF10.DCIS and MDA-MB-231 cells in 3-dimensional cultures and in mouse xenografts. Here we extend the oncogenic role of AKT3 to the preneoplastic setting and report that the regulation of AKT3 in this context by miRNA-29c appears to be of functional and biologic relevance.

### Targeting AKT that is activated during preneoplastic progression inhibits cell proliferation in MCF10.AT1 and MCF10.DCIS cells

**Figure 10:**
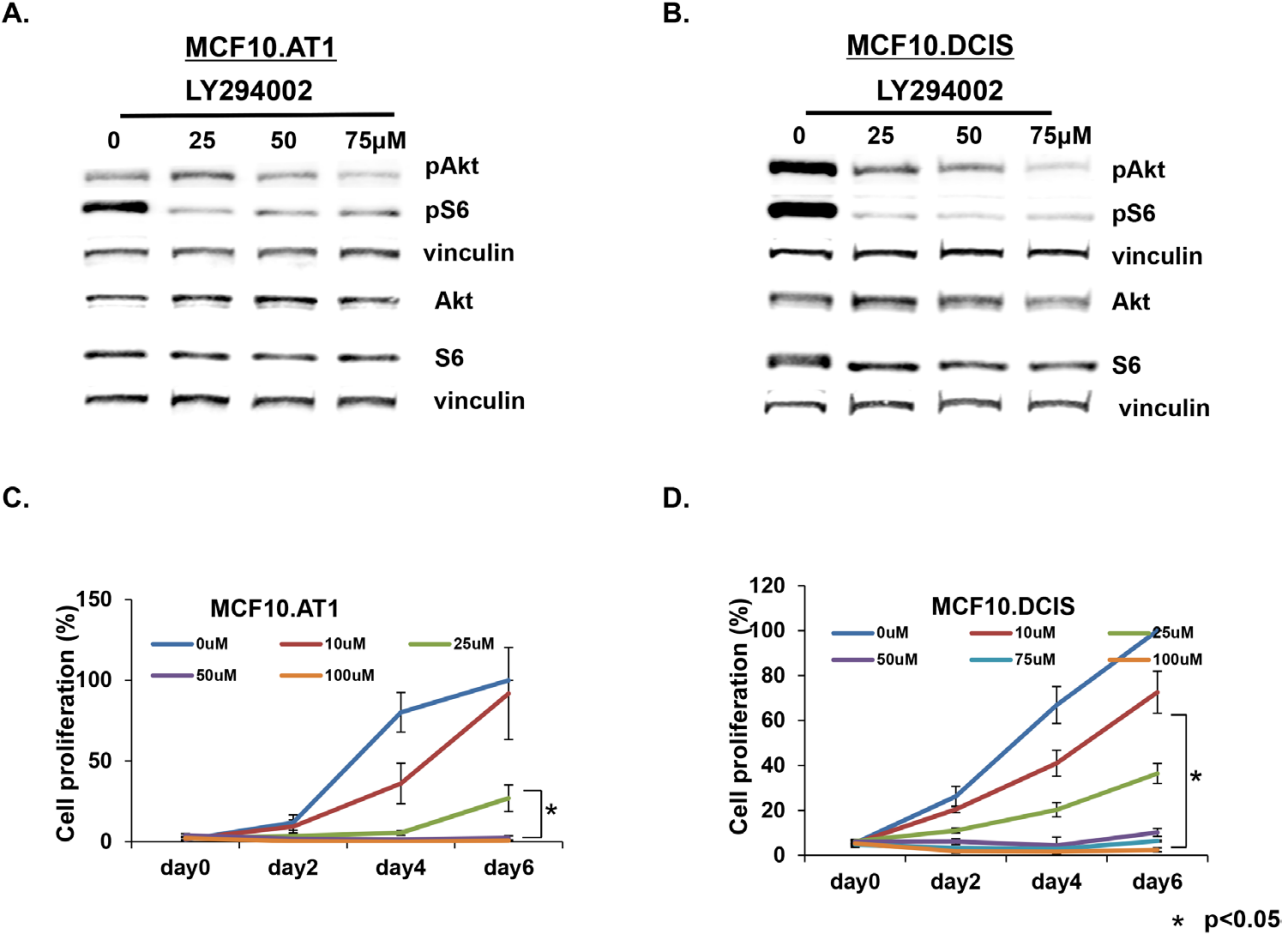
AKT-mTOR pathway targeting inhibits cell proliferation in MCF10.AT1 and MCF10.DCIS cells **A, B**. Western blots showing total and phosphorylated endogenous protein levels of AKT-mTOR pathway in vehicle- or LY294002-treated MCF10.AT1 and MCF10.DCIS cells. The cells were treated with the indicated concentrations of LY294002 for 24 hrs. **C, D**. MTT assay showing inhibition in cell proliferation of MCF10.AT1 and MCF10.DCIS cells by the PI3K inhibitor LY294002 for the indicated days and concentrations. * p<0.05.

As a proof of concept that the genes we identified through our pipeline have relevance to TNBC prevention, we targeted AKT3 using LY294002, a small molecule inhibitor of upstream PI3 kinase, and studied its effect on cell proliferation in preneoplastic MCF10.AT1 and MCF10.DCIS cells. LY294002 caused significant suppression of pAKT and pS6 (Figure 10A & 10B, Supplementary Figure 2) in both MCF10.AT1 and MCF10.DCIS cells and also significantly impaired cell proliferation (Figure 10C & 10D), indicating that the genes/pathways identified through our bio-informatics approach have relevance as potential targets for prevention. Specifically, LY294002 reduced levels of pAKT (including pAKT1/2/3) and the downstream mediator pS6 by more than 50% in both MCF10.AT1 and MCF10.DCIS cell lines (Supplementary Figure 2). LY294002 treatment at 100 μM for 6 days led to a significant inhibition (~100% in both MCF10.AT1 and MCF10.DCIS cells) in cell proliferation as well (Figure 10C & 10D). AKT pathway and cell proliferation inhibition caused by LY294002 are not TNBC subtype specific to [19, 20] and have been linked with high toxicity in patients (indicating LY294002's effects on normal cells as well) [21, 22]. Therefore, while these proof-of-principle results show that AKT pathway inhibition is growth inhibitory in preneoplastic and DCIS cells, use of available agents for targeting of this pathway for TNBC prevention is currently not feasible, mainly because of the unfavorable pharmacokinetics, poor selectivity, and toxicity concerns of this class of drugs [21, 22].

### DNA methylation plays a role in suppression of miRNA-29c

**Figure 11:**
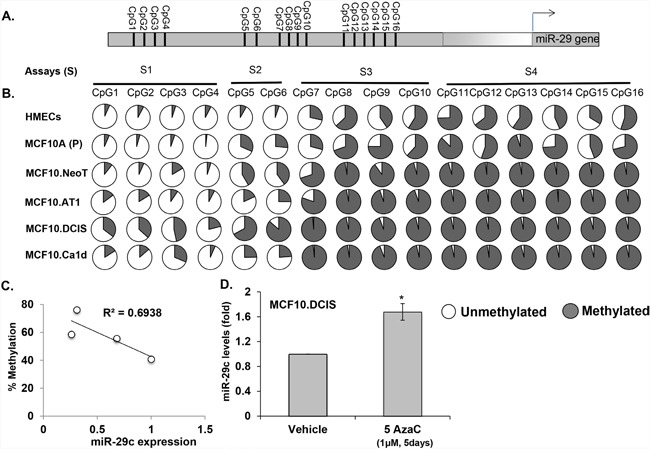
Methylation of miRNA-29c correlates with its expression during the spectrum of TNBC progression and its demethylation reverts the expression of miRNA-29c **A**. Schematic diagram of the *miRNA-29c* gene promoter showing the location of CpG sites. **B**. Percent of methylation level at each CpG site in the miRNA-29c gene promoter determined from genomic DNA obtained from an MCF10A-based TNBC progression model by pyrosequencing. The data are averages of methylation levels from 2 different cell passages. **C**. Correlation analysis showing an inverse correlation between expression of miRNA-29c and average percent methylation level of 16 CpGs in the promoter during TNBC progression. **D**. Taqman-based real-time PCR analysis of MCF10.DCIS cells treated with the DNA demethylation drug 5-aza cytadine at a 1 μM concentration for 5 days to measure miRNA-29c-3p levels that were normalized to RNU44 control.

To broaden the potential of miRNA-29c-centered strategies for TNBC prevention, we next considered the possibility of restoring miRNA-29c expression. To do so, we first explored the mechanism behind the loss of miRNA-29c in the early preneoplastic process in TNBC and studied the DNA methylation status of miRNA-29c gene promoters. We measured the methylation of 16 CpG sites in the miRNA-29c gene promoter region that, as described by Poli *et al* [23], is ~20 kb upstream of the miRNA-29c gene (the genomic coordinates and the primer sequences for studying DNA methylation are provided in Supplementary Figure 3) (Figure 11A). We found these 16 CpGs to progressively gain methylation, from being hypomethylated in non-cancerous immortalized TNBC MCF10A (P) cells to being hypermethylated in invasive MCF10.Ca1d cells (Figure 11B). The average methylation of the first 7 CpGs in this region (CpG1-7) increased gradually and progressively from a baseline methylation of 15% in MCF10A (P) cells, to ~ 26% in preneoplastic cells (MCF10.NeoT1 and MCF10.AT1) and 56% in DCIS. The methylation rate then decreased to ~ 30% in the invasive MCF10A.Ca1d cells. On the other hand, CpG8-16 started out comparatively more methylated (70%) in the non-cancerous MCF10A (P) cell line, became almost fully methylated (96%) in the preneoplastic stage, and remained fully methylated (96%) throughout the remaining progression of TNBC. As a control, we also tested the methylation levels of normal primary human mammary epithelial cells (HMECs) and found the HMEC methylation levels at all 16 CpG sites to be similar to those of non cancer, MCF10A (P) cells (Figure 11B).

Our correlation analysis in the MCF10A-based TNBC progression series showed an inverse correlation (r=0.69) between overall levels of DNA methylation of all 16 CpGs and miRNA-29c expression (Figure 11C), suggesting that DNA methylation accounts for a significant degree of the miRNA-29c repression seen during TNBC tumorigenesis. The average DNA methylation rates for all 16 CpG residues were 41% in non-cancerous MCF10A (P), 56% in preneoplastic MCF10.NeoT and MCF10A.AT1, 76% in MCF10.DCIS, and 59% in invasive MCF10.Ca1d cells.

Next, to confirm that DNA methylation of the miRNA-29c gene promoter indeed regulates the expression of miRNA-29c, we treated MCF10.DCIS cells, which had the highest methylation rate, with 1 μM 5-azacytadine (a DNA demethylating agent) or vehicle control for 5 days. The level of mature miRNA-29c relative to an internal control (small nuclear RNA RNU44) increased significantly (~70%) (Figure 11D). Collectively, these studies suggest that DNA methylation at least in part regulates miRNA-29c expression during TNBC progression. DNA methylation was previously shown to be responsible for basal-subtype-specific loss of miRNA-29c in breast cancer cell lines and in tissues of breast cancer patients [23], and strikingly, our studies have extended these findings and shown for the first time that DNA demethylation causes miRNA-29c to be lost as early as during normal to hyperplasia/atypia transition during the development of TNBC.

Lastly, it would be interesting to know the mechanisms that cause hypermethylation of the miRNA-29c gene promoter during TNBC progression. One way by which this progressive increase in DNA methylation can be achieved is through an increase in the DNA methyl transferase enzyme (DNMT) during breast cancer progression. Indeed, we found an increase in DNMT3A (which is known to be involved in both de novo and maintenance methylation of DNA) in our MCF10A-based breast cancer progression panel (data not shown). While it is plausible that this increase in DNMT3A may cause hyper- methylation of the miRNA-29c gene promoter (and thus repression of miRNA-29c) during TNBC progression, we are not aware of any reports on whether it directly binds and regulates the miRNA-29c gene promoter. Conversely, DNMT3A has been reported to be downstream of miRNA-29c and is a validated gene target of miRNA-29c in the brain [24] and breast cancers (in particular miRNA-29c-5p, our unpublished observations).

In summary, we report that expression of the tumor suppressor miRNA-29c is lost as early as the preneoplastic stage of TNBC tumorigenesis. Loss of miRNA-29c seems to be biologically relevant in basal-like breast cancer patients, where low levels of miRNA-29c predict worse overall survival. We find that in the earliest stages of TNBC development, miRNA-29c appears to exert its biologic activity through direct binding and regulation of several key mediators, namely TGIF2, CREB5, and AKT3. Lastly, our studies suggest that DNA methylation is involved in the suppression of miRNA-29c early in TNBC progression. These findings provide opportunities to develop and test novel strategies for TNBC prevention.

## MATERIALS AND METHODS

### MCF10A model system of breast cancer progression

We used the MCF10A cell line-based model system that was developed by Fred Miller and colleagues for studying breast cancer progression [25–28]. The isogenic cell lines from this model system comprise the entire range of human breast cancer progression. MCF10A (P) is an immortalized human mammary epithelial cell line; MCF10.NeoT and MCF10.AT1 represent the hyperplastic/atypical hyperplastic premalignant cell lines that are generated by HRAS transformation of MCF10A (P) cells [27]. MCF10.DCIS cells form comedo DCIS lesions in immune-deficient mice [29]. MCF10.CA1d and MCF10.CA1h cells, both derived from MCF10AT xenografts, form well-differentiated malignant tumors in xenograft mouse models. We purchased the MCF10A (P) cell line from ATCC and obtained the MCF10.AT1, MCF10.neoT, MCF10.Ca1d, and MCF10.Ca1h cell lines from the Karmanos Cancer Center, Detroit, MI, under a Materials Transfer Agreement. MCF10.DCIS cell were bought from Wayne State University, Detroit, MI. Primary HMECs were purchased from ATCC, Manassas, Virginia. All the cell lines used in the study were authenticated by the selling agency and used within the first 10 passages.

### Small RNA isolation and qPCR analysis

Total RNA, including miRNA, was extracted by using the miRNeasy Mini Kit (Qiagen, [Germantown, MD]), an extraction method that efficiently preserves the small RNA (10-200 nucleotides) fraction. RNA integrity was measured using the Agilent 2100 Bioanalyzer [Santa Clara, CA], and only samples that passed strict quality control standards were processed for next-generation sequencing performed by the Sequencing Core at Baylor College of Medicine, Houston, TX. cDNA was prepared from RNA by using the iscript cDNA synthesis kit (BioRad, [Hercules, CA]). The target mRNA levels were measured with respect to a loading control (ribosomal protein L19) by using the SYBR green-based qPCR method, as described previously [30]. The primer sequences used for all gene targets tested are in Supplementary Table 1. Levels of mature miRNA-29c were quantified by Taqman-based qPCR using the Taqman miRNA assay from ThermoFisher Scientific [Waltham, MA] and following the manufacturer's instructions.

### Next generation sequencing & analysis of functional miRNA/mRNA pairs

For small RNA library construction, which yields 25-30 million reads per library, RNA samples were prepared using the DGE-Small RNA Sample Prep Kit (Illumina, [San Diego, CA]) as described previously and were analyzed on the Illumina HiSeq2000 platform [11, 31]. mRNA sequencing yielded 30-40 million read pairs for each sample; the data were first mapped to the human genome, (build UCSC hg19) using aligner, TopHat2 [32]. Gene expressions and their differences were computed using Cufflinks [33]. A combined profile of all samples was computed and quantile normalization was applied. Supervised learning analysis was performed using the t-test statistic via the R statistical system. Genes with an increase greater than 1.25 fold were selected and run through the Gene Set Enrichment Analysis (GSEA) software (Broad Institute, [Cambridge, MA]) [34], and GSEA implementation at the Molecular Signature Database (MSigDB) was used to screen for pathways and processes [35]. We further determined for each gene the number of enriched pathways it belongs to, and sorted the genes in decreasing order of that number. To further integrate signature miRNAs and mRNAs, we employed the SigTerms methodology [11], which looks for trends in miRNA and corresponding gene target expression in breast cancer progression and also determines their strength of association. By applying a one-sided Fisher exact test, we determined the miRNAs for which the gene targets are significantly enriched (Q<0.25) in the gene signature. All the small RNA seq data generated in this study is submitted to GEO database under accession number GSE93740.

### Cell proliferation

The anti-proliferative effects of the PI3K inhibitor LY294002 on MCF10.AT1 and MCF10.DCIS cells were determined by the MTT dye uptake method. The effects of miRNA-29c mimics on the cell proliferation marker Ki67 were studied by using a Ki67 antibody-based immunofluorescence assay as described elsewhere [36, 37]. Ki67 antibody was purchased from DAKO (Agilent Technologies, Santa Clara, CA) and used at a working dilution of 1:500. The intensity of Ki67 staining representing the proliferation index of cells was measured by counting the cells that expressed high levels (> 3 foci) of Ki67 staining (Ki67-positive cells) or low levels (0-2 foci) of Ki67 staining (Ki67-negative cells).

### Clonogenic cell survival assay

The colony forming ability of the MCF10.AT1 and MCF10.DCIS cells after transfection with miRNA-29c mimics or scramble control mimic was measured by plating 500 cells/well (in a 6-well dish) in their regular media for about 12 days. At the end of 12 days, the cells were stained with 0.5% crystal violet (in methanol) for 5 minutes. Following staining, the cells were washed in water, and the plates were dried overnight. Colonies with more than 50 cells were counted as a clone.

### TCGA data mining

We evaluated the association between miRNA-29c levels and overall patient survival in the basal breast cancer dataset (TCGA, https://tcga-data.nci.nih.gov/tcga/). Basal-like breast cancer samples (n=80) used for this analysis were from the small RNA Sequencing dataset of 825 patient samples described and published by the TCGA consortium [9]. Demographic and clinical characteristics of these patients have been described elsewhere [9]. For this analysis, only the survival outcomes were used and linked to miRNA 29c expression levels. Other clinical or demographic variables were not considered or adjusted for. First, we sorted the data according to miRNA-29c expression, and then association with overall survival was evaluated by comparing the top 50% and the bottom 50% of the specimens using the log-rank test (p<0.05). Overall, survival significance was evaluated by employing the package “survival” [38] in the R statistical system.

### Western blotting

Thirty to 40 μg of total cellular protein was subjected to sodium dodecyl sulfate–polyacrylamide gel electrophoresis; transferred to Hybond ECL nitrocellulose (Amersham, [Pittsburgh, PA]); and probed with pAKT (S473), AKT, S6, and pS6 (S240/244), antibody, or the loading control, vinculin. Proteins were detected by using the “Odyssey classical Imager” Infrared Imaging System (Li-Cor Biosciences, [Lincon, NE]). We were able to probe a single Western blot membrane for more than 2 proteins of interest (of different sizes, by cutting the membrane) and vinculin because of the ability of the Odyssey system to detect signals from antibodies raised in mice and rabbits on the same membrane at separate wavelengths. As a result, in our Western blots, one common vinculin band is shown for multiple proteins if the proteins came from the same membrane.

### Cell transfections

MCF10.AT1 and MCF10.DCIS cells were transfected using Lipofectamine 2000 (Invitrogen Technologies, [Carlsbad, CA]) following the manufacturer's instructions. Cells were plated in 6-well culture dishes and then transfected with miRNA-29c-3p–mimic or scramble mimic (10 nM) with/without pmiRGLo vector containing miR-binding sites. After a 5-h incubation in OptiMEM, the medium was replaced with regular cell culture medium supplemented with 2X fetal bovine serum/horse serum. Forty-eight hours after the transfection, cells were lysed or plated for further assays.

### Cloning and luciferase assay

The wild type and the 3- nucleotide mutated seed sequence of the broadly conserved binding sites of miRNA-29c-3p (as predicted by Target Scan) and 200 flanking nucleotides (both upstream and downstream) from the AKT3, CREB5, and TGIF2 of 3′UTR regions were PCR amplified from the MCF10.AT1 genomic DNA and cloned downstream of the firefly luciferase open reading frame at the PmeI and XbaI sites by using primers (described in Supplementary Table 1) in pmiRGLo vector (Promega Corporation, [Madison, WI]). The empty vector and 3′UTR containing reporter constructs were transfected by Lipofectamine 2K (Life Technologies, [Carlsbad, CA]) in MCF10.AT1 cells. Forty-eight hours after the transfection, luciferase activity was measured by using the Dual-Luciferase reporter system (Promega, [Madison, WI]).

### Plasmids

The expression vector of the AKT3 gene containing cDNA (1440nt mRNA sequence, aka 1236 pcDNA3 Myr HA Akt3) (Addgene plasmid no. 9017) was a gift from Dr. William Sellers [Dana-Farber Cancer Institute]. The TGIF2 expression vector (containing the mRNA sequence only) that was generated from an EST (GenBank™ accession no. AW411096.1), and was a gift from Dr. David Wotton [University of Virginia] [39]. The expression vector containing complete cDNA of CREB5 in a pCMV-GFP backbone was purchased from MyBioSource [San Diego, CA]. The expression vector containing complete cDNA of wild-type CDK6 (981nt) in a pCMV-neo-Bam backbone was created by Dr. Martha Grossel [Connecticut College] and was a generous gift to us [40]. These expression vectors contained cDNA sequences without 3′UTR or 5′ UTR; therefore, the biological effects observed by overexpression of these expression vectors were the result of direct overexpression of these oncogenes and not a consequence of miRNA-29c binding to any regulatory regions in the 3′UTR.

### Pyrosequencing DNA methylation analysis

Two μg of the genomic DNA was treated with sodium bisulfite using the EZ DNA methylation-gold kit (Zymo Research, [Irvine, CA]) according to the manufacturer's instructions and was subsequently used for pyrosequencing analysis. These analyses were performed at the DNA Methylation Analysis Core, The University of Texas MD Anderson Cancer Center. PCR primers for studying the miRNA-29c gene promoter were designed using the Pyromark Assay Design SW 1.0 software (Qiagen, [Germantown, MD]). The genomic coordinates of the region studied and the primer sequence to analyze 16 CpG sites in this region are shown in Supplementary Figure 3). Several controls, including high methylation (SssI-treated DNA), low methylation (WGA-amplified DNA), and no-DNA template controls, were included in each assay. The assay design, PCR, and sequencing of the PCR products were performed as described elsewhere [41]. The degree of methylation was calculated using the Pyro-Q CpG 1.0.9v software (Biotage AB, [Sweden]).

### Statistical analyses

Student's unpaired t test was applied to all the data except MTT experiments to calculate the level of significance (p value) between various groups studied. MTT data were analyzed by using the Kruskal-Wallis method followed by Dunn's post hoc test. Experiments were conducted 3 separate times, and the values shown represent the mean ± standard error of mean. P values <0.05 were considered significant.

## SUPPLEMENTARY MATERIALS FIGURES AND TABLES



## Abbreviations

TNBC: triple-negative breast cancer
TCGA: The Cancer Genome Atlas
DCIS: ductal carcinoma *in situ*
AT: atypia
ADH: atypical ductal hyperplasia
AKT3: V-Akt murine thymoma viral oncogene homolog 3
TGIF2: TGFB-induced factor homeobox 2
CREB5: CAMP-responsive element binding protein 5
CDK6: cyclin-dependent kinase 6
PI3K: phosphoinositide 3-kinase.
