# Supplementary material for: Regulation of miRNA-29c and its downstream pathways in preneoplastic progression of triple-negative breast cancer
Source: Oncotarget. 2017 Jan 30;8(12):19645–60. doi: 10.18632/oncotarget.14902 (PMC5386711; doi:10.18632/oncotarget.14902)
Supplement: Supplementary file 1 [file oncotarget-08-19645-s001.pdf]

Regulation of miRNA-29c and its downstream pathways in preneoplastic progression of triple-negative breast cancer

SUPPLEMENTARY FIGURES AND TABLE

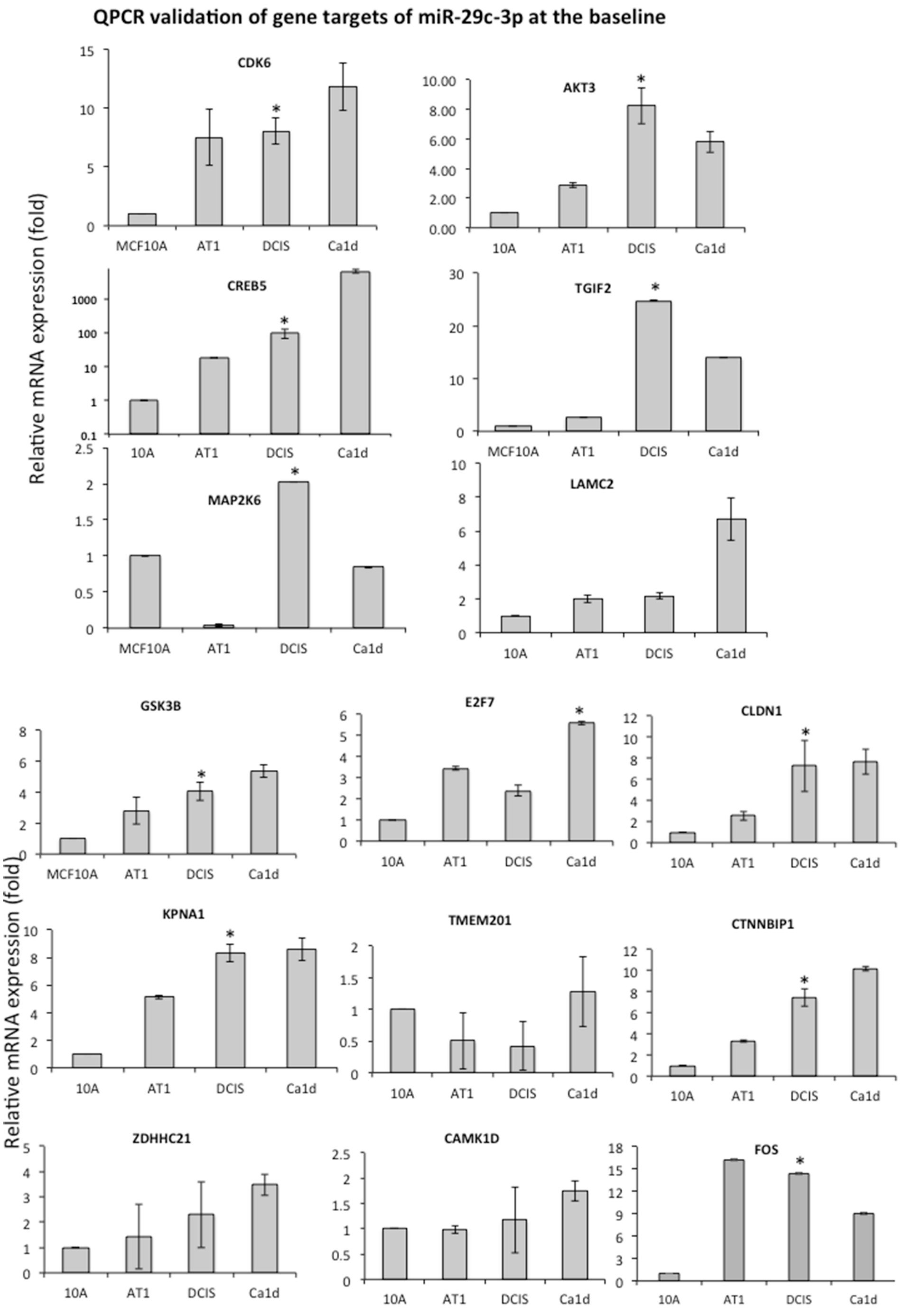

Supplementary Figure 1: QPCR validation miRNA-29c-3p gene targets at baseline. PCR analysis of indicated miRNA-29c-3p gene targets measured in MCF10A based TNBC progression model, normalized to L19 control. Values represent data generated from three different experiments, and is plotted as mean ± standard error.

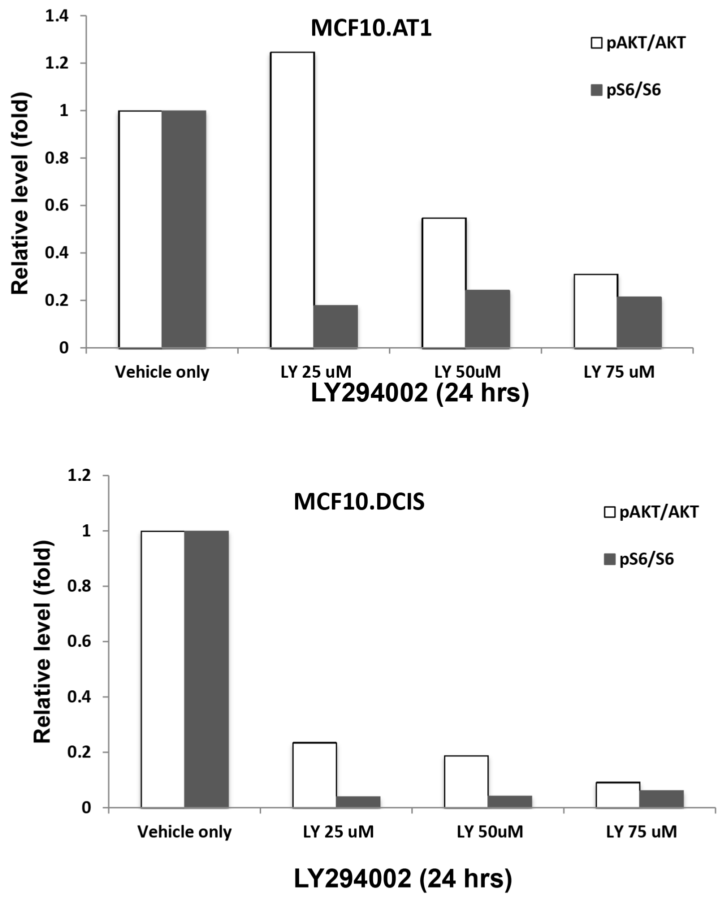

Supplementary Figure 2: AKT-mTOR pathway targeting inhibits cell proliferation in MCF10.AT1 and DCIS cells. Quantification of western blot (Figure 10) showing total and phosphorylated endogenous protein levels of AKT-mTOR pathway in vehicle or LY294002 treated MCF10.AT1 and MCF10.DCIS cells.

Genomic coordinates of the miR-29c gene promoter for studying DNA methylation:
>hg19_dna range=chr1:207995584-207997869 5'pad=0 3'pad=0 strand=+ repeatMasking=none

Primer sequences to study methylation of miR-29c gene promoter:
Forward primer: 5'GATCCCAGCAAATACAT3'
Reverse primer: 5'TGGGTAGCCTAGGCTAG3'
Length of the region studied- 274bp
Sequence of the region analyzed: GATCCCAG*CAAATACATACGTGGGGGAAGAAGGGGGTTACGCCATCAAGTCCTGAAGCCCGTCG GACCACCCATCGCCGCCTGCGCAGACCCAAATCTTGGTCCCGCCGTAAGGTGCCGCAGTCCCGAATGTTCCAGAATTTGGTCCCATCAAA CCCTCCACCGTCGCCCCACAACCTCTTGCTCCCACCCCTGCCCCCCACCACCACCCCACCT*CCTCCCCACGGGAACCGCCCGTGCCACCTTG CGTGTCATCTCCTAGCCTAGGCTACCCA
Color coding key: Forward PMA primer, Reserve PMA primer, S1 assay probe ,S2 assay ,S3 assay, S4 assay

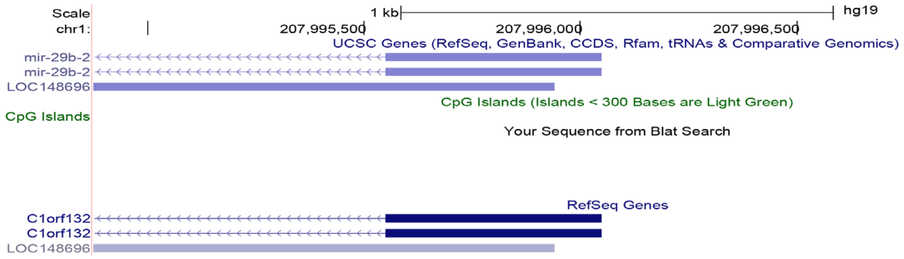

Supplementary Figure 3: Genomic coordinates of the miRNA-29c gene promoter for studying DNA methylation. The genomic coordinates of miRNA-29c gene promoter are shown using human genome built 19. Forward and reverse primer sequence and the sequence of the region for studying DNA methylation are also provided.

Supplementary Table 1: Primer sequences

**QPCR Primers**		
**Gene**	**Primer F**	**Primer R**
AKT3 F1R1	CCAAATAAACGCCTTGGTGGA	CTTGGCTGGTCTGGGATGTC
ZDHHC21 F1R1	ACCAAAGCGTTCCCATCACT	AGGATCTTCCAACGAGTGCC
CDK6 F2R2	TGCACAGTGTCACGAACAGA	CGTGACGACCACTGAGGTTA
CREB5 F2R2	GACGAGGATCCGGACGAGA	TCCAATGACATCACCCAGACC
CTNNBIP1 F2R2	GGAAGAGTCCGGAGGAGATGT	TTCCGTCTCCGACCTGGAAA
FOS F2R2	CCGGGGATAGCCTCTCTTAC	GTGGGAATGAAGTTGGCACT
GSK3B F2R2	CAAACAGACGCTCCCTGTGA	TCCTGACGAATCCTTAGTCCA
RPS6KA3 F1R1	ACAGCGCTGAGAATGGACAG	CCGAACTCGGTCTCGAACTT
MAPK10 F1R1	ATGTGGAGAATCGGCCCAAG	GCACCTGTGCTGAAGGAGAA
E2F7 F1R1	ACCAGCCTTCAAGTGGATCG	GGTCCGTCTGAGCAACAGA
LAMC2 F1R1	CTGTCACTGGAGAACGCTGT	ACATCTTGATGGCGCTGTGA
TGIF2 F1R1	TACAACGCCTACCCCTCAGA	GCCCTGGTCAGCAGAGTAAG
CAMK1D F2R2	GTCCATCGGAGTGATTGCCT	TGCTGCCGAGGTGTAGTTTT
RPS6KA3	GCAGAAGATGGCTGTGGAGA	AAGCTGCCTAGCATCAGAGC
SPRY4	CCGGCTTCAGGATTTACACA	CACATGGCTGGTCTTCACCT
KPNA1 F1R1	TGCATTTGCTGAGTAGCCCA	CCAGTGCCATTCCTTTTGGC
**3' UTR cloning primers**		
CREB5 (1-427bp)	TAGTgtttaaactaaatgcaccatcagacctgg	TAGTtctagatacggtatgggaaaggctgtgtg
TGIF2 (1-550bp)	TAGTgtttaaactagcatctgccaagaagggtgc	TAGTtctagatacctgggctaactactgccgtgaatgc
AKT3 (1401-1900bp)	TAGTgtttaaactactttacgtggcaaaatgaac	TAGTtctagatacatctgcatatgaatatgattc
**Site directed mutagenesis primers**		
CREB5 SDM	GGAAACGCTTGGGAGATTTTCTCCAGTTTTC	GAAAACTGGAGAAAATCTCCCAAGCGTTTCC
TGIF2 SDM	GAGACAAGCAGGGAGATGCTGCTTCTGC	GCAGAAGCAGCATCTCCCTGCTTGTCTC
AKT3 SDM	GGTCTCATGCTCTTCGTGCTACTGTC	GACAGTAGCACGAAGAGCATGAGACC
